# Fabrication of Cobalt-Nickel-Zinc Ternary Oxide Nanosheet and Applications for Supercapacitor Electrode

**DOI:** 10.3389/fchem.2018.00597

**Published:** 2018-11-29

**Authors:** Chun Wu, Lei Chen, Xuechun Lou, Mei Ding, Chuankun Jia

**Affiliations:** ^1^College of Materials Science and Engineering, Changsha University of Science and Technology, Changsha, China; ^2^Key Laboratory of Advanced Energy Materials Chemistry, Ministry of Education, Nankai University, Tianjin, China

**Keywords:** Co-Ni-Zn ternary oxide, electrode materials, nanosheet, supercapacitor, energy storage

## Abstract

Mesoporous cobalt-nickel-zinc ternary oxide (CNZO) nanosheets grown on the nickel foam are prepared by a simple hydrothermal treatment and subsequent calcination process. The physical characterizations show that the as-obtained CNZO nanosheets possess the mesoporous structure and a high specific surface of 75.4 m^2^ g^−1^ has been achieved. When directly applied for the binder-free supercapacitor electrode for the first time, the nickel foam supported mesoporous CNZO nanosheet electrode exhibits an ultrahigh specific capacity about 1172.2 C g^−1^ at 1 A g^−1^. More significantly, an asymmetric supercapacitor based on the as-obtained CNZO positive electrode and an activated carbon negative electrode shows a high energy density of 84.2 Wh kg^−1^ at a power density of 374.8 W kg^−1^, with excellent cycle stability (keeps 78.8% capacitance retention and 100% coulombic efficiency after 2,500 cycles). The excellent supercapacitive properties suggest that the nickel foam supported CNZO nanosheet electrodes are promising for application as high-performance supercapacitor.

## Introduction

Supercapacitors have been wildly investigated in recent years because of their advanced properties including long cycle stability, fast charge/discharge rate, high power density and environment-friendly and is becoming one of the promising energy storage system (Jia et al., 2015; Ding et al., 2018; Wang et al., 2019; Zhang et al., 2018b). Meanwhile, they also play an essential role to solve the serious environmental and energy crisis. Among different types of the supercapacitors, the faradaic capacitors with energy storage mechanisms based on the redox reactions have attracted considerable attention, which exhibit much higher specific capacitance than that of electrical double-layer capacitors (EDLCs) (Zeng et al., 2019). Accordingly, develop high-performance transition metal oxides and/or conducting polymers as faradaic capacitor electrode material with abundance in resources, low price and facile and scalable preparation process seems to be particularly important.

Recently, the transition metal oxides, such as MnO_2_, NiO, Co_3_O_4_, et al. have been extensively explored due to the advantages including easy large-scale fabrication and excellent flexibility in morphology and structures (Cheng et al., 2014; Wu et al., 2018; Cui et al., 2019). Particularly, mixed transition metal oxides with two or three metal ions are emerging as promising electrode materials thanks to several advantages compared to the simple transition metal oxides, such as enhanced electronic conductivity (because of multiple oxidation states based on different metal ions for reversible faradaic reactions), high electrochemical activities, and relatively low activation energy for electron transfer between cations. However, several drawbacks of low intrinsic conductivity, big volume change and slow ion diffusion rates during the electrochemical measurement process, limit the applications of the transition metal oxides for high-performance supercapacitor.

Studies illustrate that the electrode materials directly grown on the conductive substrates such as carbon cloth and nickel foam and applied as the binder-free electrodes can significantly improve the electrochemical performance of the supercapacitors. Specifically, the well-designed three-dimensional (3D) hierarchical architectures grown on the substrates with advantages of large specific surface areas resulting in reactive sites and better permeabilities of the electrolyte, have been employed for practical applications in supercapacitors, lithium ion batteries, and other energy storage devices (Li et al., 2015; Zhao et al., 2017; Zhang et al., 2018a). A supercapacitor electrode of 3D self-supported Co_3_O_4_@CoMoO_4_ core-shell architectures on nickel foam has been prepared by Wang et al. The Co_3_O_4_ nanocones grown vertically on the nickel foam are served as the core and CoMoO_4_ nanosheets developed on the surface of the nanocones are acted as the shell. High specific capacitance, good rate capability, and cycling stability can be achieved (Wang et al., 2016). Via a facile strategy, Zhang et al. synthesized hierarchical Co_3_O_4_@NiCo_2_O_4_ nanowire array nanoforests. The smart combination of Co_3_O_4_ and NiCo_2_O_4_ nanostructures in this hybrid architectures presents a promising synergistic supercapacitive effect with greatly improved properties. The electrode exhibits a high areal capacitance of 2.04 F cm^−2^ at 5 mV s^−1^ and 0.79 F cm^−2^ even at 30 mA cm^−2^. Significantly, when the current turned back to 10 mA cm^−2^ after long cycles with high current density, a areal capacitance of 1.18 F cm^−2^ can be recovered, which can remain for another 1,500 cycles with excellent stability (Zhang et al., 2013). Another study about the supercapacitor electrode of 3D NiCo_2_O_4_@Co_x_Ni_1−x_(OH)_2_ core-shell nanosheet arrays have been rationally designed and facilely synthesized on the nickel foam via an electro-deposited method. Electrochemical measurements show that a large areal capacitance about 5.71 F cm^−2^ at 5.5 mA cm^−2^ can be achieved when the mass loading of the as-prepared NiCo_2_O_4_@Co_x_Ni_1−x_(OH)_2_ electrode material is about 5.5 mg cm^−2^. Furthermore, it also shows an excellent rate capability (Xu et al., 2014). The efficient electron and ion transportation, large surface areas resulting in easy electrolyte access to electrode and good strain accommodation makes 3D substrate supported hierarchical architectures attracting much attentions (Mohana Reddy et al., 2012; Gu et al., 2019; Wu et al., 2019). It can not only achieve high contact between substrate and electrode active materials resulting in great improvement of ion transportation, but also provide channels for the transportation of the charge. The above properties are beneficial to maximize the utilization of electrochemically active material. Therefore, simple processes for design and fabrication of mixed transition metal oxides with desired properties are needed to be exploited urgently.

Herein, a facile and environmental-friendly approach to synthesize 3D mesoporous CNZO nanosheet grown on nickel foam has been reported and the physical and electrochemical properties as binder-free electrode material for supercapacitors have been characterized. The mixed ternary metal oxides are supposed to offer a synergistic effect contributed by zinc, nickel, and cobalt ions of the mixed metal oxides on redox reactions, relating to the reasons that the electrical conductivity and capacitive performance can be enhanced by Zn, which possess good electrical conductivity; The active site density and conductivity can be improved by Ni, which shows high capacity. Meanwhile, Co can provide enhanced electronic conductivity. In addition, multi-phase metal oxides would be generated by incorporating various metal ions (Liu et al., 2013). All these would be benefit for the electrochemical performance improvement of the electrode.

The physical characterizations show that the as-obtained CNZO nanosheets possess the mesoporous structure and a high specific surface of 75.4 m^2^ g^−1^ has been achieved. When directly applied for the binder-free supercapacitor electrode for the first time, the nickel foam supported mesoporous CNZO nanosheet electrode exhibits an ultrahigh specific capacity about 1172.2 C g^−1^ at 1 A g^−1^. More significantly, the binder-free CNZO electrode reveals low internal resistance (*R*_s_) and interfacial charge transfer resistance (*R*_ct_) values. The excellent supercapacitive properties suggest that the nickel foam supported CNZO nanosheet electrodes are promising for application as high-performance supercapacitor.

## Experimental

### Synthesis of CNZO nanosheet material

The nickel foam supported CNZO nanosheet material is prepared by a simple hydrothermal treatement and a followed calcination process. The nickel foam with 3.5 × 6 cm size was washed and dried for the reaction. 12 mmol urea (CO(NH_2_)_2_), 4 mmol ammonium fluoride (NH_4_F), 3 mmol nickel sulfate hexahydrate (NiSO_4_·7H_2_O), 6 mmol cobalt sulfate heptahydrate (CoSO_4_·7H_2_O), and 3 mmol zinc sulfate heptahydrate (ZnSO_4_·6H_2_O) were mixed with 80 mL DI water. The mixed solution and the treated nickel foam were kept at 130°C for 5 h after transferred into a 100 mL Teflon-lined stainless steel autoclave. The sample was kept in an ultrasound bath and washed with distilled water for several times and dried at 60°C for 12 h. Finally, put the as-obtained samples into the furnace and treated in air atmosphere at 350°C for 2 h to achieve the nickel foam supported CNZO nanosheet material. The mass of the CNZO materials on the nickel foam is about 1.6 mg cm^−2^.

### Characterization of material

Field emission scanning electron microscopy (FESEM Hitachi S4800) was used to gain the microstructure and morphology information of the as-prepared CNZO sample. By using X-ray diffraction (Rigaku SmartLab XRD), the crystalline structure of the CNZO sample can be achieved. Furthermore, to investigate the microstructure of the CNZO sample, a Quantachromeautosorb automated gas sorption system was employed and adsorption/desorption isotherm of nitrogen was tested.

### Electrochemical measurements

In the electrolyte of 6 M KOH solution, a three-electrode system with CNZO electrode acting as working electrode, nickel foam serving as counter electrode and Hg/HgO applying as reference electrode was employed to study the electrochemical performances of CNZO electrode. All of the supercapacitive characterizations, including cyclic voltammetry measurements (CV), galvanostatic charge/discharge measurement as well as electrochemical impedance spectroscopy (EIS), were conducted on a electrochemical workstation system (CHI660). The cycle stability of the supercapacitor is measurement under the Neware battery testing system.

## Results and discussion

### Structure analysis

**Figure 1:**
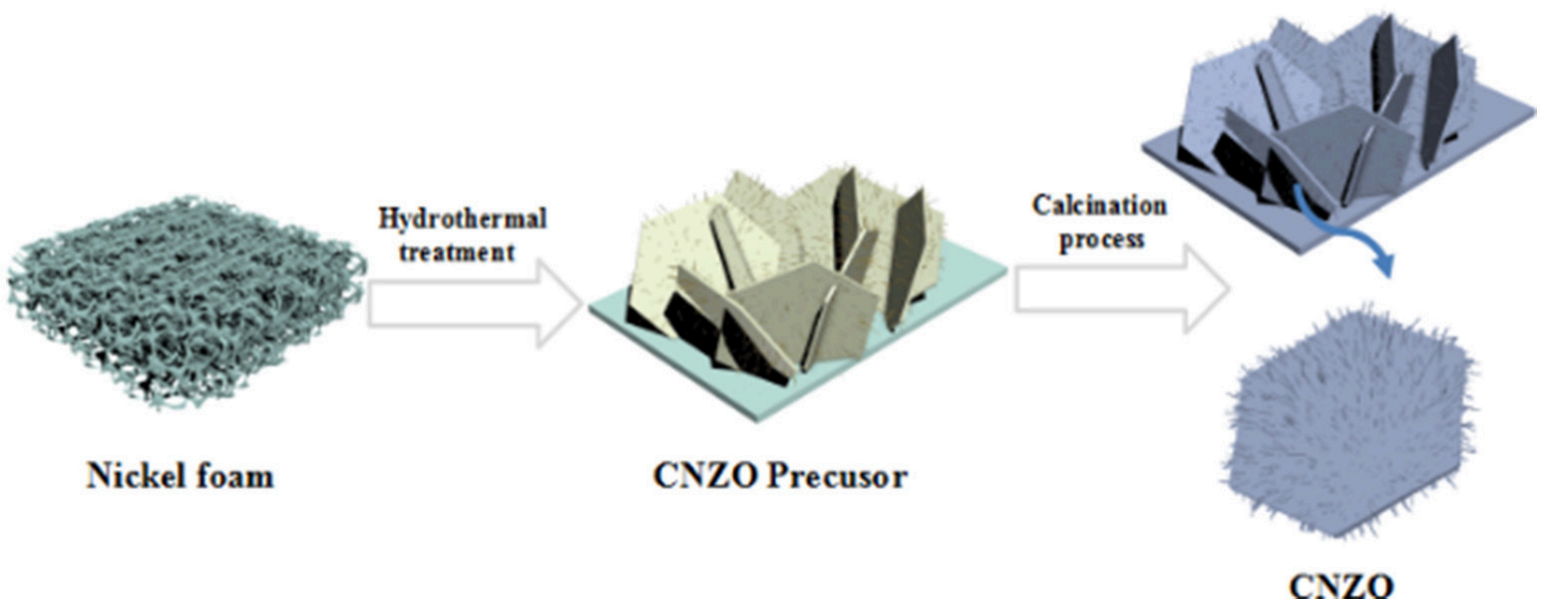
Illustration of the preparation process of the CNZO nanosheet materials.

Figure 1 illustrates the preparation process of the mesoporous CNZO nanosheet electrode materials. First, by hydrothermal treatment under 130°C for 5 h, the raw mixed solutions react and the precursor of mesoporous CNZO nanosheet materials have been synthesized. Then, via a calcination process in air atmosphere under 350°C for 2 h, the as-obtained CNZO nanosheet precusor transforms into the final mesoporous CNZO nanosheet electrode material. The growth of the mesoporous CNZO nanosheet on the current collector would result in direct contact between the as-prepared active material and the substrate, thus lead to the short electrons and ions diffusion path and achieve very low contact resistance, which are beneficial for the electrochemical performance improvement of the as-obtained CNZO electrode.

**Figure 2:**
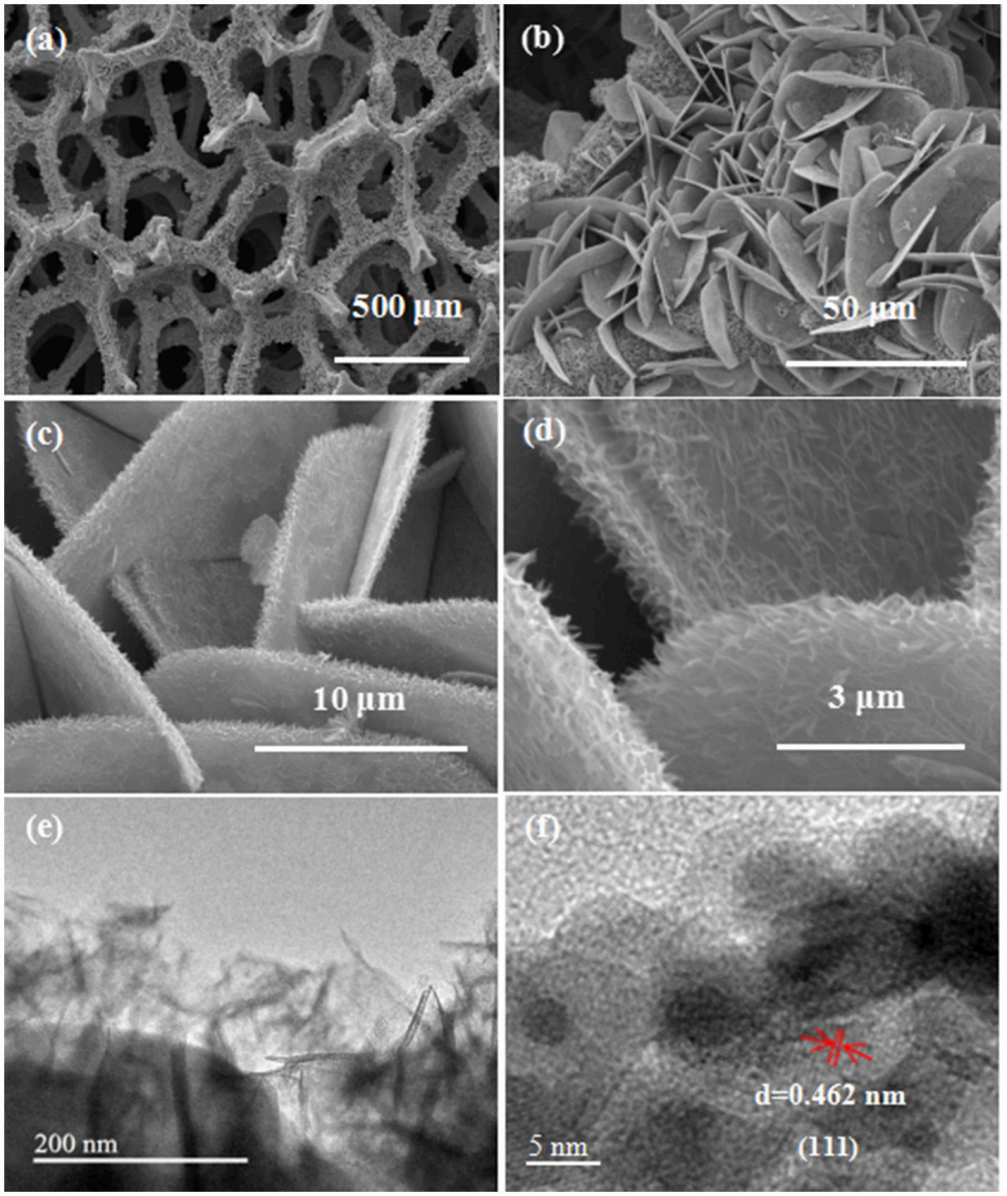
**(a–d)** SEM images **(e,f)** TEM images of the as-prepared mesoporous CNZO nanosheet material.

The observation of microstructure and morphology for the as-prepared mesoporous CNZO nanosheet material has been investigated by FESEM. It can be notably noticed from Figures 2a,b of the low magnification SEM image that numerous nanosheets homogeneously grow on the surface of the nickel foam to form a 3D nanostructure. The diameter of the nanosheet is about 20–40 μm, and the thickness is about 0.4 μm measured from the high magnification SEM image of Figures 2c,d. More interestingly, it also can be seen that numerous burr-like structures on the surface of the nanosheets, which would lead to larger contact area between electrode material and electrolyte compared to the pure nanosheet and result in excellent electrochemical behaviors. The microstructure of the as-prepared electrode material is further observed by TEM and present as follows. It can be noted that the porous structure is existed in the edge part of the nanosheet, which constructed from numerous nanoparticle subunits (see Figure 2f). Highly resolved lattice fringes with a measured interplanar spacing of 0.462 nm is also shown, which corresponds to the (111) plane of the spinel Co-based metal oxide phase (Xiao et al., 2014).

**Figure 3:**
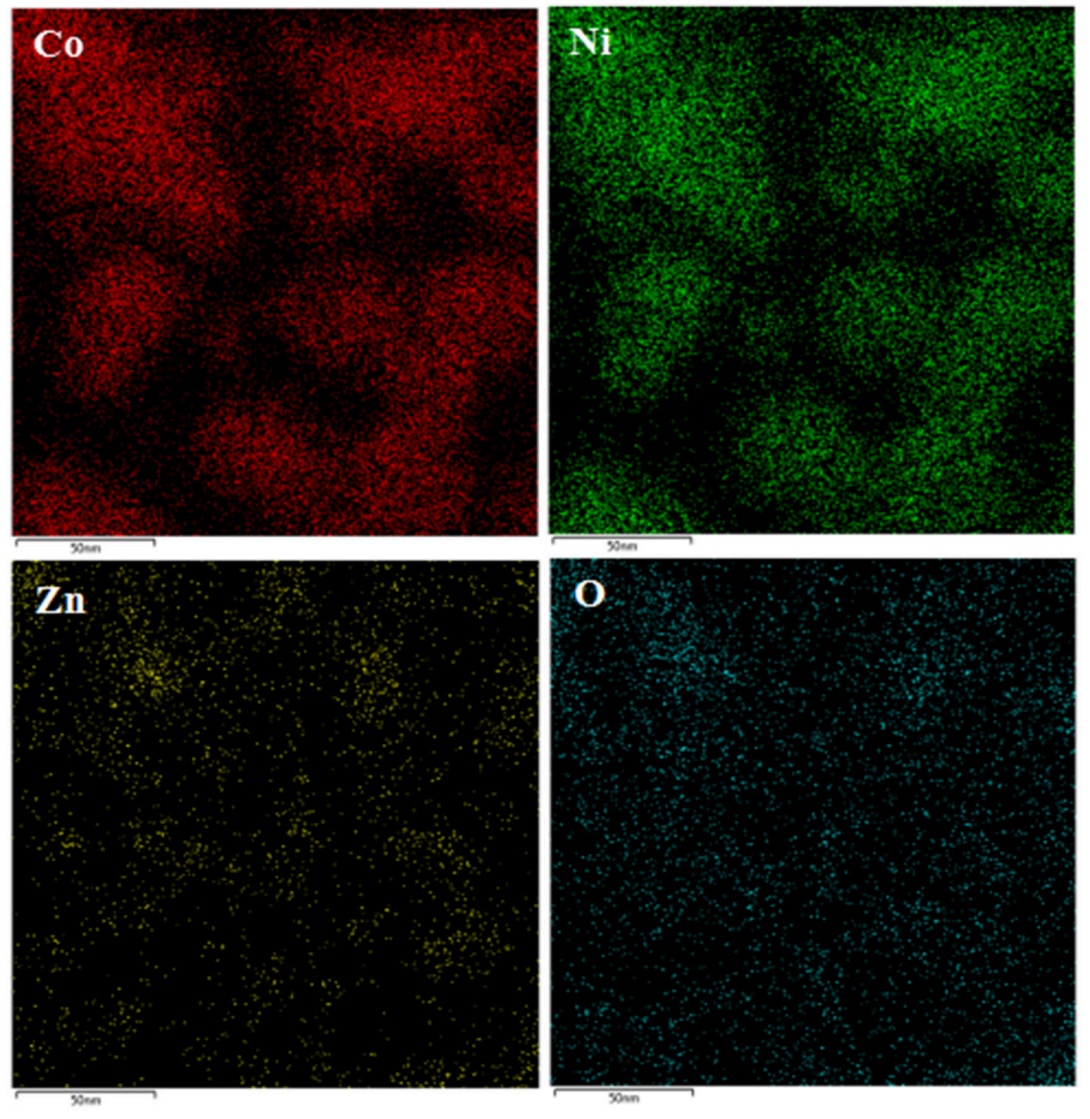
EDS mapping images of the as-prepared mesoporous CNZO nanosheet material.

Via the elemental mapping analysis under SEM observation, the elemental distribution of the as-prepared CNZO nanosheets can be observed. Figure 3 displays the corresponding element mapping results, which apparently presents the existence of the Co, Ni, Zn, and O and their homogeneous distribution, indicating the successful formation of ternary metal oxide CNZO electrode material.

**Figure 4:**
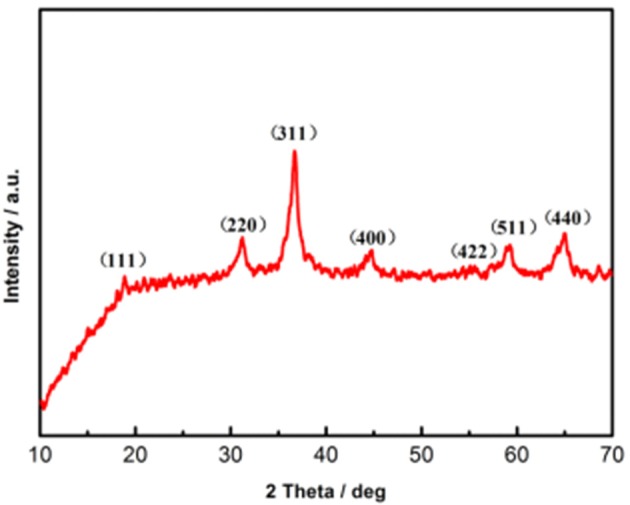
XRD pattern of the as-prepared mesoporous CNZO nanosheet material.

After being scraped off from the substrate to eliminate effects originated from the nickel foam, the crystal structure of CNZO nanosheet material power is characterized by XRD analysis. All the diffraction peaks of the CNZO sample in Figure 4 can be indexed to the the spinel Co_3_O_4_ (PDF No. 74-2120) with slightly shift. The reason can be ascribed to the differences of the metal ionic radii of Ni, Co, and Zn. However, the substitution of Ni and Zn element does not significantly affect the crystal structure of spinel Co_3_O_4_ (Li et al., 2014). Besides, no additional peaks for other phases can be observed.

**Figure 5:**
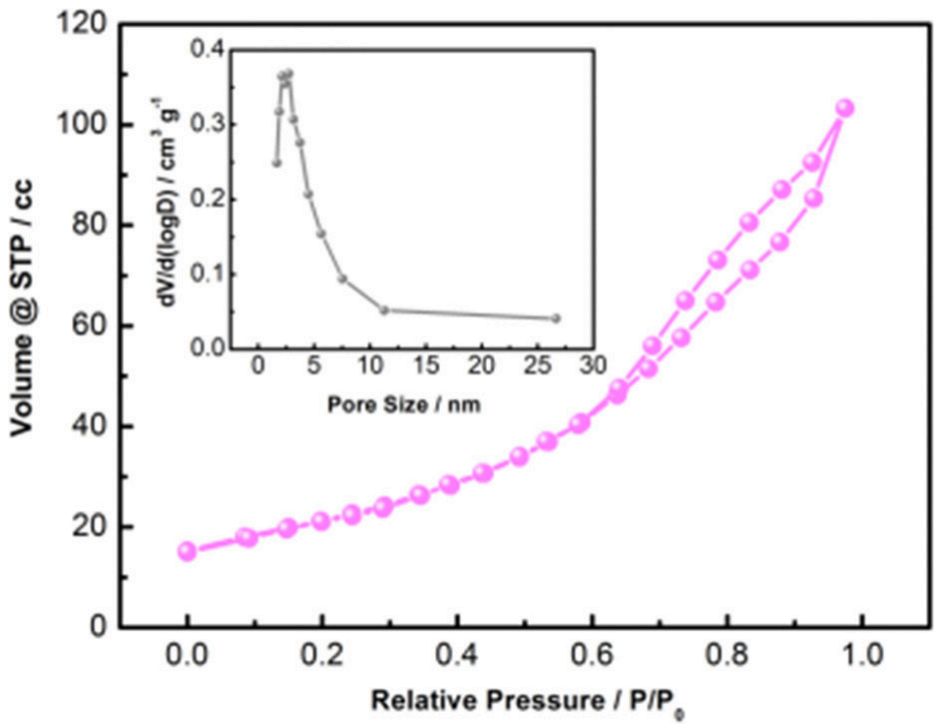
N_2_ absorption-desorption isotherm of the hierarchical mesoporous CNZO electrode materials, the inset is the pore distribution.

The investigation of the as-prepared mesoporous CNZO nanosheet microstructures is conducted via the nitrogen adsorption measurement. Figure 5 exhibits the pore size distribution curves and nitrogen adsorption-desorption isotherms. Evidently, the curve of the nitrogen adsorption-desorption isotherm for the as-prepared CNZO material is close to that of IUPAC type-IV with distinct hysteresis loops (Rojas et al., 2002), suggesting the mesoporous structure of the as-prepared sample. The pore size distribution of the as-prepared CNZO material is presented in the inset curve, it can be seen that the pore size is centered at about 2.5 nm, which further confirms the presence of the mesopores in this CNZO nanosheet material. The BET specific surface area of the as-prepared mesoporous CNZO nanosheet material is measured to be about 75.4 m^2^ g^−1^. With large specific surface area, the nickel foam supported CNZO nanosheet material would bring about more reactive sites and result in better penetration of the electrolyte into the whole electrode materials during the electrochemical measurement process, all these would be beneficial for the excellent supercapacitive properties.

**Figure 6:**
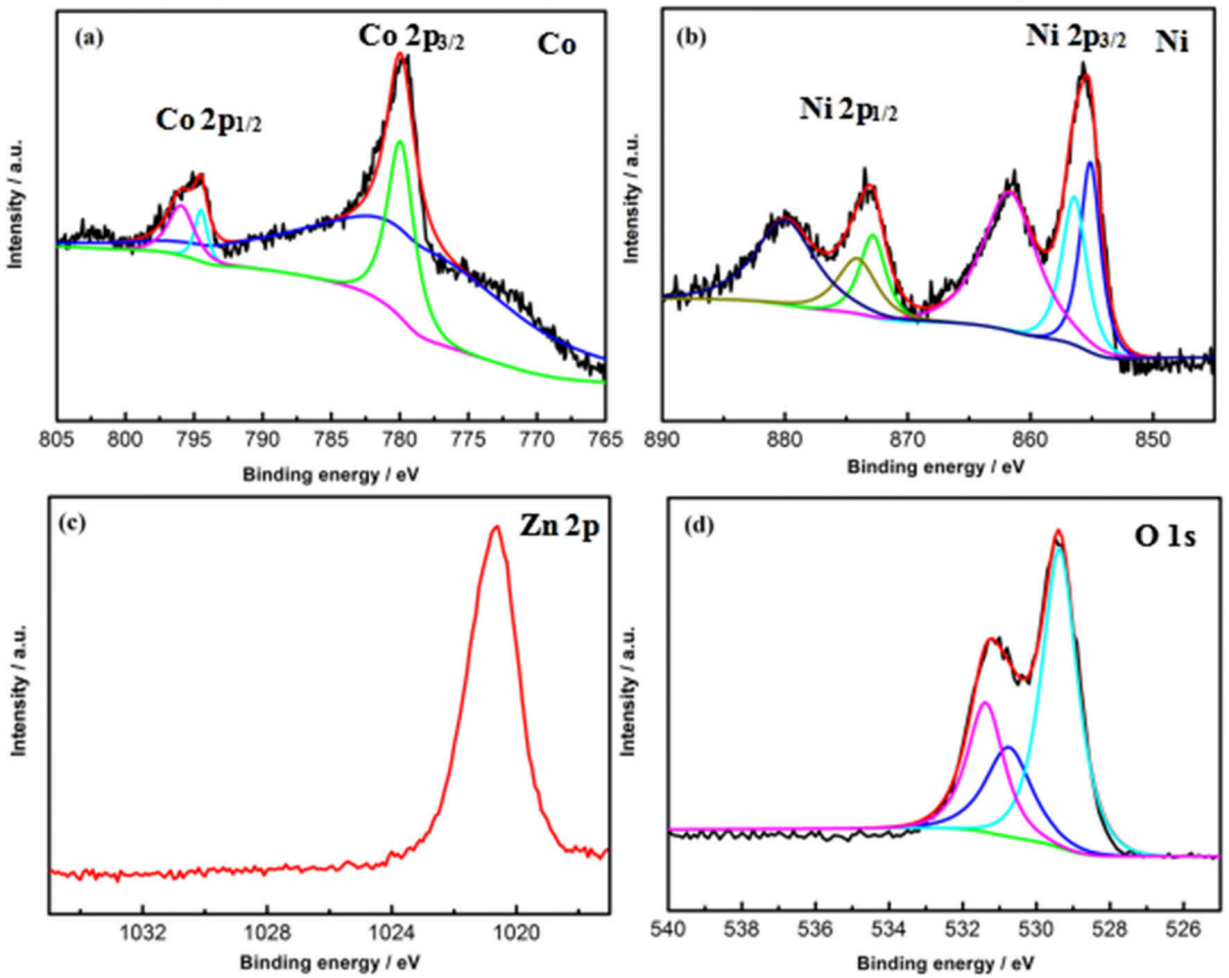
XPS of CNZO materials: high-resolution of **(a)** Co2p, **(b)** Ni2p, **(c)** Zn2p, and **(d)** O1s spectra.

X-ray photoelectron spectroscopy is utilized to characterized the elemental composition and oxidation state of the electrode material. Figure 6 shows the high-resolution of Co, Ni, Zn, and O spectra. As depicted in Figure 6a, two distinguished doublets located at a high-energy (Co 2p1/2) and low-energy band (Co 2p3/2) can be observed in the high-resolution of Co 2p spectrum. The spin-orbit splitting value of Co 2p1/2 and Co 2p3/2 is over 15 eV, indicating the presence of Co^3+^and Co^2+^ (Xiong et al., 2015). Meanwhile, the high-resolution of Ni 2p spectrum in Figure 6b can be fitted with two shakeup satellites and two spin-orbit doublets, which shows the characteristic of Ni^3+^ and Ni^2+^ (Xiong et al., 2015). Besides, as for the high-resolution of Zn 2p spectrum in Figure 6c, a major peak at 1020.6 eV can be seen, which can be attributed to the Zn 2p3/2 of Zn(II). Furthermore, two peaks at 531.2 and 529.3eV of the O 1s can be noted from Figure 6d, corresponding to the oxygen species contained in the CNZO material. The above results demonstrates that the Co^3+^, Co^2+^, Ni^3+^, Ni^2+^, and Zn^2+^ are existed in the CNZO material.

### Electrochemical behaviors of the CNZO electrode

**Figure 7:**
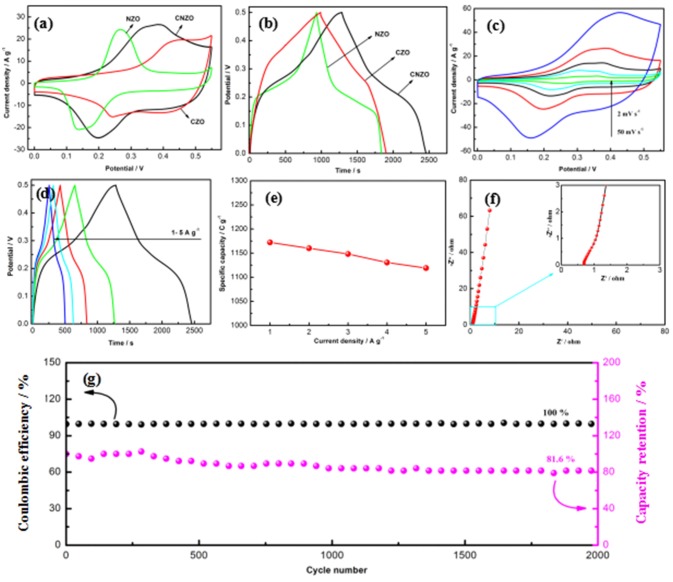
**(a,b)** CV and charge-discharge curves of CNZO, CZO, and NZO electrodes, **(c)** CV curves of CNZO at various scan rates, **(d)** charge/discharge curves of CNZO electrode at various current densities, **(e)** the rate performance of the CNZO electrode under different current densities, **(f,g)** Nyquist plot and cycle life of the CNZO electrode.

CV measurement under the potential window from 0 to 0.55 V is employed to investigated the supercapacitive behaviors of the CNZO electrode. Figure 7a present the CV curves of the NZO, CZO, and CNZO electrodes at 20 mV s^−1^, which shows that the CNZO electrode exhibits the largest increment in the CV integrated area, suggesting the largely enhanced electrochemical reaction activity of the CNZO electrode material. Figure 7b displays the charge/discharge curves of the NZO, CZO, and CNZO electrodes at 1 A g^−1^, it can be seen that the CNZO electrode exhibits the longest discharge time and thus delivers the highest specific capacitance. The CV curves of the CNZO electrodes at the scan rates from 2 to 50 mV s^−1^ (see Figure 7c). It can be evidently seen that the shapes of all the CV curves exhibit typical faradaic behaviors. A pair of redox peaks can be clearly observed in the CV curves, indicating that the electrochemical behaviors of CNZO electrode generate from their faradaic reactions. The energy storage mechanism of the as-prepared CNZO electrode can be attributed to the Faradic redox reactions assigned to the M-O/M-O-OH (M stands for both Co and Ni ions) (Wang et al., 2011). Furthermore, with the increase of the scan rates, the redox peak positions shift progressively, this can be ascribed to the existence of polarization (Wu et al., 2012). Moreover, the specific capacity can be calculated based on the CV curves according to the following equation (1) (Brousse et al., 2015):

(1) $$\begin{array}{l} C=\frac{Q}{m}=\int \frac{idt}{m} \end{array}$$

Here *i* represents a sampled current value (A), *dt* stands for a sampling time span (s), and m is the mass of the CNZO materials (g). The maximum specific capacity of the CNZO electrode is calculated to be about 1185.1 C g^−1^ at the scan rates of 2 mV s^−1^.

The supercapacitive behaviors of the CNZO electrode can be further studied by the charge/discharge measurement and the charge/discharge curves under 1–5 A g^−1^ are exhibited in Figure 7d. Obviously, all curves are symmetric in shape, indicating the excellent electrochemical behaviors of the CNZO electrode. Moreover, the specific capacity can be calculated based on the charge/discharge curves according to the following equation (2) (Brousse et al., 2015):

(2) $$\begin{array}{l} C=i_{m}^{*}\Delta t \end{array}$$

Where, *i*_*m*_ (A g^−1^) is the current density and Δ*t* is the discharge time (s). The specific capacity of the CNZO electrode is calculated to be about 1172.2 C g^−1^ at 1 A g^−1^. And the specific capacities under various current densities are exhibited in Figure 7e, excellent rate capability with 95.4% capacity retention can be achieved even at a high current density of 5 A g^−1^. The high specific capacity and wonderful rate capability suggests that the CNZO electrode can be a very promising materials for the applications of supercapacitor.

To further study the supercapacitive performance of the as-prepared CNZO electrode, the EIS measurement with amplitude of 5 mV ranging from 10^5^ to 10^−2^ Hz is operated (see in Figure 7f). The curve exhibits a straight line in the low frequency region and a small semicircle in the high frequency region. The straight line can be attributed to the presence of the Warburg impedance, which displays the result of the frequency dependence of ion diffusion to the electrode interface (Zhang et al., 2012). In the high frequency region from the inset curve, the first intersection point shows the internal resistance (*R*_s_) and the diameter of the semicircle stands for the interfacial charge transfer resistance (*R*_ct_) (Qiu et al., 2015). As can be noted that the *R*_s_ and *R*_ct_ values of CNZO electrode are very small, demonstrating the excellent electrochemical performance of the CNZO electrode.

The cycle stability of the electrode material is an essential parameter for the supercapacitor application, and Figure 7g presents the cycle behavior of the CNZO electrode under the current density of 5 A g^−1^. It can be obviously noted that after 2,000 cycles, the capacity retention is about 81.6% and the coulonbic efficiency remains 100%, suggesting the excellent cycle performance of the CNZO electrode.

**Figure 8:**
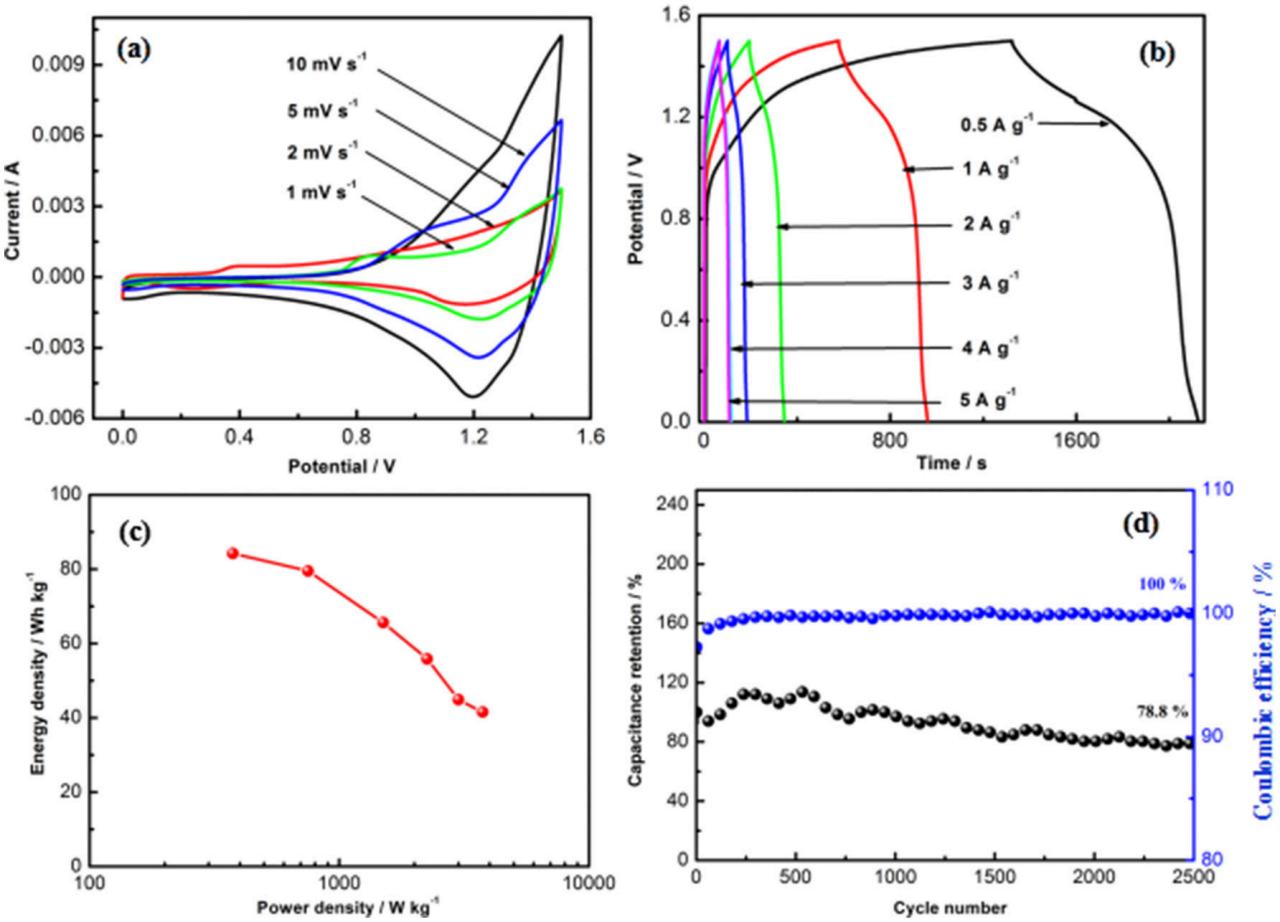
**(a)** CV curves of the CNZO//AC ASC device at different scan rates, **(b)** charge/discharge curves of the CNZO//AC ASC device under different current densities, **(c)** Ragone plots of the CNZO//AC ASC device, **(d)** capacitance retention and coulombic efficiency during the charge/discharge cycle of the CNZO//AC ASC device at 5 A g^−1^.

To further evaluate the practical supercapacitors application of the CNZO electrode material, an asymmetric supercapacitor (CNZO//AC ASC device) based on the CNZO positive electrode and the active carbon (AC) negative electrode has been fabricated. The electrochemical performance of the AC electrode is reported in the previous work (Wu et al., 2015). The supercapacitive behaviors of the CNZO//AC ASC device is conducted during the potential window range from 0 to 1.5 V in 6 M KOH solution. Figure 8a displays the typical CV curves of the CNZO//AC ASC device under the scan rates of 1–10 mV s^−1^. A pair of well-defined redox peaks can be seen from the CV curves between the potential window at 0.8 and 1.5 V, indicating battery-type behavior of the as-fabricated CNZO//AC ASC device. Meanwhile, the corresponding charge/discharge curves under the current densities of 0.5–5 A g^−1^ are shown in Figure 8b, it can be seen that the discharge curves are slightly non-linear during the potential window, which is in agreement with the above CV measurements. The maximum specific capacitance of the CNZO//AC ASC device is calculated to be 269.5 F g^−1^. As we all known the the energy and power density are the vital parameters for the supercapacitor application, which can be calculated based on the charge/discharge measurements of the CNZO//AC ASC device according to the following equations (Wu et al., 2015):

(3) $$\begin{array}{l} E=\frac{1}{2}CV^{2} \end{array}$$

(4) $$\begin{array}{l} P=\frac{E}{\Delta \text{t}} \end{array}$$

where *E* represents the energy density of the device (Wh kg^−1^), *C* is the specific capacitance of the device (F g^−1^), *V* shows the potential window (V), *P* is power density of the device (W kg^−1^), Δ*t* stands for the discharge time (s). A Ragone plot for the as-fabricated ASC device is presented in Figure 8c, which shows an high energy density of 84.2 Wh kg^−1^ at a power density of 374.8 W kg^−1^ and energy density of 41.6 Wh kg^−1^ even at a power density of 3.8 kW kg^−1^. The electrochemical behaviors are better than those of the previous reports, such as Co_3_O_4_//AC (Zhang et al., 2014), NiCo_2_O_4_//AC (Zhang et al., 2015), ZnCo_2_O_4_//AC (Guan et al., 2014), NiWO_4_//AC (Niu et al., 2013), and CoMoO_4_//AC (Yu et al., 2014). Furthermore, the cycle performance of the CNZO//AC ASC device under the current density of 5 A g^−1^ is shown in Figure 8d, which presents that the capacitance retention keeps 78.8% and the coulombic efficiency remains 100% after 2,500 cycles, demonstrating the excellent cycle stability of the as-fabricated CNZO//AC ASC device.

The wonderful electrochemical behavior of the CNZO electrode can be ascribed to the following aspects: First, the synergistic effect among different metal element that Ni ion can make a contribution of the high capacity, Co and Zn ions may provide enhanced electronic conductivity, may result in improved capacitive property of the CNZO electrode. Second, the nickel foam supported CNZO nanosheet could eliminate use of polymer binders, thus result in a very low “dead volume” and lead to high interfacial contact between the substrate and the active materials, these would improve the ion transportation and achieve excellent supercapacitive behaviors. Third, the mesoporous CNZO nanosheet material with highly porous structures and large specific surface area may significantly bring about more reactive sites and result in better penetration of the electrolyte into the whole electrode materials during the electrochemical measurement process, all these would be beneficial for the excellent supercapacitive properties.

## Conclusions

The nickel foam supported mesoporous CNZO nanosheet electrode material has been successfully prepared by a simple hydrothermal treatment and subsequent calcination process. The physical characterizations show that the as-obtained CNZO nanosheets possess the mesoporous structure and a high specific surface of 75.4 m^2^ g^−1^ has been achieved. When directly applied for the binder-free supercapacitor electrode for the first time, the nickel foam supported mesoporous CNZO nanosheet electrode exhibits an ultrahigh specific capacity about 1172.2 C g^−1^ at 1 A g^−1^. More significantly, an asymmetric supercapacitor based on the CNZO nanosheet positive electrode and an activated carbon negative electrode shows an high energy density of 84.2 Wh kg^−1^ at a power density of 374.8 W kg^−1^, with excellent cycle stability (keeps 78.8% capacitance retention and 100% coulombic efficiency after 2,500 cycles). With the excellent supercapacitive properties, the nickel foam supported CNZO nanosheet electrode could be employed for the high-performance supercapacitor electrodes and other energy storage system.

## Author contributions

CW designed and performed the experiments. CW, LC, and XL prepared the samples and analyzed the data. CW, MD and CJ participated in interpreting and analyzing the data. All authors read and approved the final manuscript.

### Conflict of interest statement

The authors declare that the research was conducted in the absence of any commercial or financial relationships that could be construed as a potential conflict of interest.
